# mHealth Apps for the Self-Management of Low Back Pain: Systematic Search in App Stores and Content Analysis

**DOI:** 10.2196/53262

**Published:** 2024-02-01

**Authors:** Tianyu Zhou, David Salman, Alison McGregor

**Affiliations:** 1 Department of Surgery and Cancer Imperial College London London United Kingdom; 2 Department of Primary Care and Public Health Imperial College London London United Kingdom

**Keywords:** smartphone, mHealth, mobile health, low back pain, self-management, treatment interventions, mobile phone

## Abstract

**Background:**

With the rapid development of mobile health (mHealth) technology, many health apps have been introduced to the commercial market for people with back pain conditions. However, little is known about their content, quality, approaches to care for low back pain (LBP), and associated risks of use.

**Objective:**

The aims of this research were to (1) identify apps for the self-management of LBP currently on the market and (2) assess their quality, intervention content, theoretical approaches, and risk-related approaches.

**Methods:**

The UK iTunes and Google Play stores were initially searched for apps related to the self-management of LBP in May 2022. A repeat search in June 2023 was conducted to ensure that any relevant new apps developed in the last year were incorporated into the review. A total of 3 keywords recommended by the Cochrane Back and Neck Group were used to search apps “low back pain,” “back pain,” and “lumbago.” The quality of the apps was assessed by using the 5-point Mobile App Rating Scale (MARS).

**Results:**

A total of 69 apps (25 iOS and 44 Android) met the inclusion criteria. These LBP self-management apps mainly provide recommendations on muscle stretching (n=51, 73.9%), muscle strengthening (n=42, 60.9%), core stability exercises (n=32, 46.4%), yoga (n=19, 27.5%), and information about LBP mechanisms (n=17, 24.6%). Most interventions (n=14, 78%) are consistent with the recommendations in the National Institute for Health and Care Excellence (NICE) guidelines. The mean (SD) MARS overall score of included apps was 2.4 (0.44) out of a possible 5 points. The functionality dimension was associated with the highest score (3.0), whereas the engagement and information dimension resulted in the lowest score (2.1). Regarding theoretical and risk-related approaches, 18 (26.1%) of the 69 apps reported the rate of intervention progression, 11 (15.9%) reported safety checks, only 1 (1.4%) reported personalization of care, and none reported the theoretical care model or the age group targeted.

**Conclusions:**

mHealth apps are potentially promising alternatives to help people manage their LBP; however, most of the LBP self-management apps were of poor quality and did not report the theoretical approaches to care and their associated risks. Although nearly all apps reviewed included a component of care listed in the NICE guidelines, the model of care delivery or embracement of care principles such as the application of a biopsychosocial model was unclear.

## Introduction

Low back pain (LBP) is a complex multifactorial disorder, often considered a combination of physical, psychological, and social dysfunction [1]. A multidisciplinary self-management intervention based on a biopsychosocial model holds significant potential to manage LBP [2,3] and has been demonstrated to be more effective than unimodal exercise therapy [4]. The National Institute for Health and Care Excellence (NICE) guidelines recommend using self-management for LBP, which can be described as the patient’s proactive adoption of strategies to manage their symptoms and monitor their health and well-being [5]. Despite being a promising approach to managing LBP, it can be challenging for an individual to self-manage any long-term condition [6]. The adherence to self-management strategies is commonly poor, especially without support, feedback, and positive reinforcement [7,8]. A qualitative study also noted poor adherence to advice and exercises as a limiting factor to recovery from LBP [9].

With the increasing popularity of electronic products, digital health solutions such as smartphone apps can be used as an innovative way to support self-management for many conditions, including LBP and may provide a solution to some of the problems outlined above [6,10]. Mobile health (mHealth) apps for pain management may be beneficial to patients [11,12], helping monitor those with acute or chronic pain and providing them with information and support for pain management. However, while many mHealth apps have been introduced into the commercial marketplace to manage pain, most have not been regulated in a uniform or standardized way before being released to the market [12,13]. The involvement of health care professionals in their development and content has been lacking [14]. This has raised concerns about the quality of these mHealth apps and whether their content information is evidence-based [15,16]. It is therefore desirable to assess the quality of current apps in the UK market and whether their content aligns with guideline recommendations. Another concerning issue is the paucity of evidence on specific intervention approaches in developed mHealth apps that have been developed, including underpinning evidence and theory [17] and relative risk management [18]. This potentially impacts on safety and efficacy of health-related smartphone apps [19].

Since there is no unified framework for assessing the theoretical and risk-based approaches associated with LBP self-management applications, we developed a theoretical framework that considers the theoretical care model of the intervention, the personalization of care, and the rate of intervention progression, as well as a risk-related framework that includes the targeted age group and the provision of appropriate safety checks. Interventions based on specific theoretical frameworks are known to be more effective in health care [20]. The Medical Research Council (MRC) guidelines strongly recommend using theoretical approaches in designing complex interventions [21], thus its inclusion in the evaluation. A critical factor in mHealth apps is personalization, where the management or treatment of the disease is tailored to the patient’s situation and individual needs, which will make the user feel that it is relevant and meaningful to them [22,23], thus the inclusion of criteria in relation to personalization. Treatment methods such as exercise need to be both adaptable and progressive. This means starting with basic exercises and gradually advancing to more complex levels at a pace that patients with LBP can comfortably handle. Such a progression helps enhance their functional abilities and quality of life, while simultaneously equipping them with strategies to effectively manage their pain. [24]. Since the treatment paths for LBP vary according to different age groups, including children, adults, and the elderly [25], the target user age group must be defined. Finally, patient safety is an essential component of health care provision and is critical to primary care management, which will effectively reduce the clinical risks associated with LBP management [18,26].

Therefore, this app review, aimed to first, identify apps for the self-management of LBP currently on the market and second, to assess their quality (eg, functionality and design), intervention content (compliance with best practice guidelines), underlying theory (eg, theoretical care model), and risk-related approaches (eg, the age group targeted).

## Methods

### Search Strategy

Apps currently on the market for the self-management of LBP were identified, reviewed, and analyzed using a systematic approach. The UK official app stores for both Apple’s iOS and Google’s Android OS were used to search for mobile apps. These 2 operating systems currently dominate the marketplace of mobile medical apps [27]. We logged into Apple iTunes and Android Google Play stores in May 2022. A total of 3 keywords recommended by the Cochrane Back and Neck Group [28] were used to search apps “low back pain,” “back pain,” and “lumbago.” A subsequent search in June 2023 was repeated to ensure that any relevant new apps in the last year were incorporated into the review.

### Study Selection

Criteria for inclusion in the review were (1) apps were a self-contained product (ie, did not depend on an external device or add-ons), (2) apps offered at least 1 active treatment option for LBP (eg, unsupervised exercise program or patient education), (3) apps only designed for people with LBP, (4) apps created or updated in the last 5 years to ensure software functionality and ongoing technical support, and (5) apps developed in English. Exclusion criteria were (1) apps targeted at managing general chronic pain, (2) apps only focused on risk factors and diagnostic tests for LBP, (3) apps only focused on specific LBP pathologies (eg, lumbar disk herniation), (4) apps designed for clinicians, (5) general back fitness apps with no mention of physiotherapy or physical therapy or musculoskeletal (MSK) conditions, and (6) apps were not downloadable or had restrictions (eg, requiring an activate access password). Apps that incurred a cost were also included; however, when both paid and free versions of an app were available, we reviewed only the paid version to ensure access to the full content. If the same app was available on iOS and Android, the iOS version was kept for inclusion and analysis.

An independent reviewer initially screened the eligible apps based on the apps’ names and descriptions and the screenshots provided. After the preliminary screening phase, the same independent reviewer downloaded apps that met the eligibility criteria for a second screening. Concerns regarding inclusion were discussed and resolved within the research group (AM and DS) until a final decision was reached.

### Data Extraction

The selected apps were downloaded onto either a Samsung SM-N975F (Android version 7.1.2) or an iPhone 12 (iOS version 16.5) for a complete assessment of eligibility and characteristics. Relevant background information offered in the included apps was extracted, including the name, version, developer, update date, cost, and presence of in-app purchases. We extracted the age or content rating and consumer rating (5-star rating system) when available. In addition, we extracted if the apps contained advertisements and whether these adverts were relevant to their back pain. It was also noted if the apps were asynchronous or synchronous (available with support from a provider), and whether the apps tracked user engagement. The collection of personally identifiable information by apps and whether consent is stated were also checked. The category of management content, specific intervention component, theoretical care model, personalization of care, the rate of intervention progression, the age group targeted, and safety checks were also extracted.

### App Content Assessment

Main categories and specific components of LBP app management content were identified and classified. Frequency analysis was performed to determine the number of apps providing these intervention contents. The recently updated 2020 NICE guideline for LBP was used to assess whether the included apps provided evidence-based interventions (categorized as “yes/no”) [29]. For this, we mapped app interventions to recommendations listed in the NICE guidelines. This guideline provides the most recent best practice recommendations for assessing and managing LBP and sciatica in people aged 16 years or older [5].

### App Quality Assessment

The Mobile App Rating Scale (MARS) was used to assess the quality of included apps in this review. MARS is a brief tool with a 23-item questionnaire to classify and assess the quality of mHealth apps for researchers, professionals, and clinicians [30]. It assesses app quality across 4 domains: engagement, functionality, aesthetics, and information quality. All items are assessed on a 5-point scale (1=inadequate, 2=poor, 3=acceptable, 4=good, and 5=excellent). The MARS has demonstrated excellent internal consistency and interrater reliability for evaluating the quality of mHealth apps [30]. To standardize the quality ratings, the assessor completed a MARS video training recommended by the developers of MARS [30]. A total of 10 apps were randomly selected for training until a consensus on the scores was reached.

### App Assessment of the Theoretical and Risk-Related Approaches

A total of 5 aspects considered in our theoretical and risk-related approaches were assessed. These included the underpinning LBP management theory, tailoring of content, the intervention of progression approach, the age group the app targeted, and appropriate safety checks.

### Ethical Considerations

This study does not involve human participants.

## Results

### App Selection

**Figure 1:**
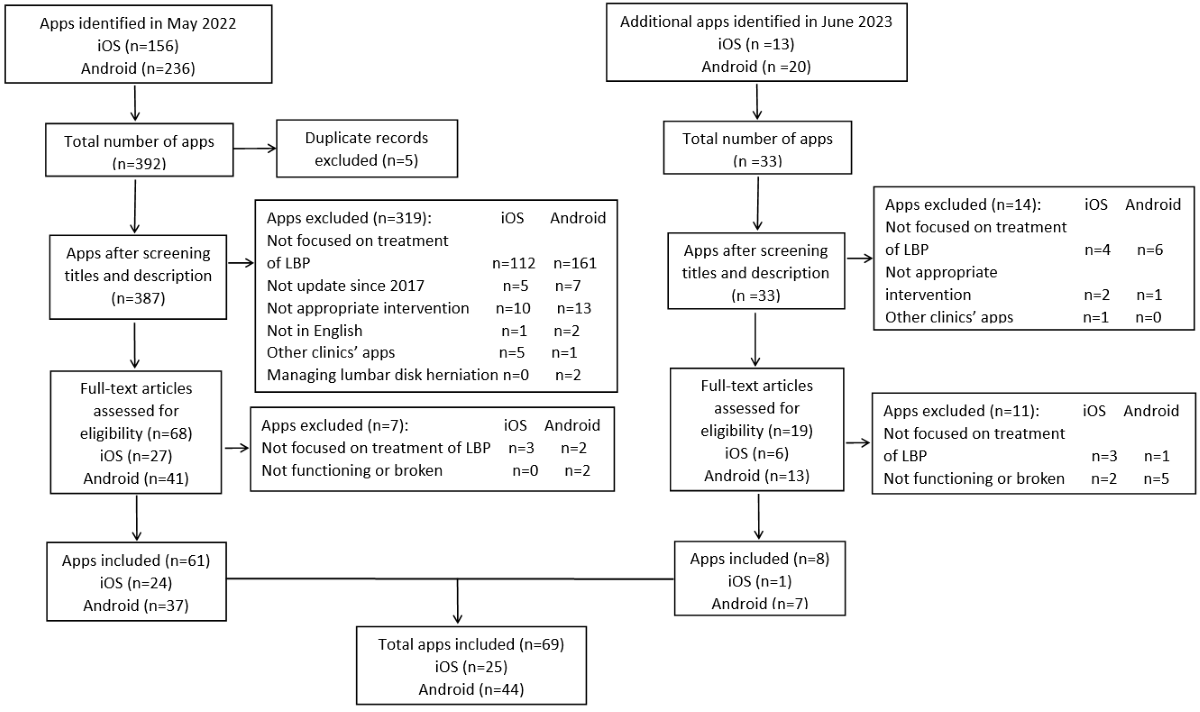
The flowchart of the app selection process. LBP: low back pain.

The searches performed in May 2022 yielded 392 apps from 2 platforms. Of these, 156 apps were identified from iTunes stores, and 236 apps were identified from Google Play stores. A total of 5 duplicates were removed, resulting in 387 apps identified for screening based on the titles and app descriptions. After initial screening, 319 apps were excluded. The eligible 68 apps were downloaded for a full evaluation and further 7 apps were excluded. The subsequent search in June 2023 found a total of 33 newly developed apps based on the initial search, of which 8 apps were newly developed. Finally, 69 apps were included in this review, of which 25 were iOS apps and 44 were Android apps. Figure 1 illustrates the selection procedure of smartphone apps for LBP.

### Characteristics of Included Apps

Of the 69 apps included in this review, 25 (36%) were found on iTunes exclusively, 44 (64%) on Google Play exclusively, and 5 (7%) were found on both app stores. There are 5 (7%) of 69 apps that required payment, ranging in price from US $1.13 to US $22.87 (median US $8.17). The majority (n=64, 93%) of the apps included were free of charge. Of these, 8 offered in-app purchases ranging from US $3.80 to US $12.70. Android apps (n=41, 93%) were more often free to access full functionality than iOS apps (n=15, 60%). Of the 41 apps reviewed by customers on a 5-star rating system, the median customer rating in 19 apps from iTunes (4.3 stars) was higher than in 22 apps from Google Play (4.1 stars). With respect to age or content rating, most of the included iOS apps (n=19, 76%) were downloaded without any age limitation: 4 apps were restricted to those of 12 years or above and 3 apps were restricted to those of at least 17 years old. However, all Android apps were labeled as suitable for all age groups.

In terms of developers, there is a mix of some health care groups and other private companies. A total of 24 (35%) apps contained advertisements, 10 (42%) of which were for products or medical companies targeting MSK disorders, leaving 14 (58%) random advertisements. In addition, all apps were asynchronous which means that they failed to deliver continuously updated application data to users. A total of 9 (13%) apps collect personally identifiable information from users and only 2 (22%) asked for their consent for collection. It appeared that none of the apps tracked user engagement. The characteristics of each app are presented in Multimedia Appendix 1.

### MARS Quality Assessment

The mean MARS total score obtained from 69 applications was 2.4 out of 5 (SD 0.44). Table 1 summarizes the MARS total scores for each app. All apps were initially assessed using MARS for engagement, functionality, aesthetics, and information. Mean scores for each subscale (out of 5) were calculated. Of the 5 categories, apps scored highest in the functionality (mean 3.0, SD 0.55) domain, followed by aesthetics (mean 2.6, SD 0.61) and engagement (mean 2.1, SD 0.58). The information domain received the lowest score (mean 2.1, SD 0.46). The MARS total score and domain score for each app are shown in Multimedia Appendix 2.

**Table 1:** Mobile app rating scale scores (N=69 apps).

MARS^a^ subscale	iOS, mean (SD)	Android, mean (SD)	Total, mean (SD)
Engagement	2.4 (0.52)	1.9 (0.55)	2.1 (0.58)
Functionality	3.1 (0.57)	3.0 (0.49)	3.0 (0.55)
Aesthetics	2.9 (0.57)	2.4 (0.47)	2.6 (0.61)
Information	2.3 (0.49)	1.9 (0.40)	2.1 (0.46)
MARS overall score^b^	2.6 (0.43)	2.3 (0.35)	2.4 (0.44)

^a^MARS: Mobile App Rating Scale.

^b^Average of 4 objective subscales.

### Intervention Contents for LBP

The LBP interventions embedded with the included apps were mainly classified into 3 categories (Table 2). Of these, most (n=47, 68.1%) of the apps offered only an exercise program, while 14.5% (n=10) apps provided patient education alone, and 13.0% (n=9) apps recommended patient education in combination with an exercise program. The remaining 2.9% (n=2) apps provided some psychological intervention for LBP in combination with an exercise program. Finally, only 1.4% (n=1) app-prescribed combinations of patient education, exercise, and psychological intervention.

**Table 2:** Number and percentage of category for low back pain interventions used in included apps.

Main category	Value, n (%)
Patient education + exercise program + psychological intervention	1 (1.4)
Exercise program + patient education	9 (13.0)
Exercise program + psychological intervention	2 (2.9)
Patient education only	10 (14.5)
Exercise program only	47 (68.1)

More specifically, of the 69 apps included in this review, most (n=51, 73.9%) apps recommended muscle stretching as a self-management strategy for LBP. A total of 42 (60.9%) apps suggested muscle strengthening, 32 (46.4%) apps offered core stability exercises, and 19 apps (27.5%) recommended using yoga to manage LBP. Also, there were 17 (24.6%) apps providing information about LBP mechanisms, followed by advice to use medication (n=9, 13%), staying active (n=9, 13%), postural therapy (n=8, 11.6%), cold and heat therapy (n=8, 11.6%), and aerobic exercise (n=8, 11.6%). Only some apps mentioned manual therapy (n=4, 5.8%), cognitive behavioral therapy (n=4, 5.8%), meditation (n=4, 5.8%), mindfulness (n=3, 4.3%), McKenzie exercise (n=3, 4.3%), electrotherapy (n=3, 4.3%), acupuncture (n=3, 4.3%), and lifestyle advice (n=2, 2.9%). Concerning app intervention content, 14 (78%) of 18 interventions for LBP were aligned with the NICE guidelines and 4 included interventions that were not endorsed by the NICE guideline: postural therapy, electrotherapy, cold and heat therapy, and acupuncture [29]. Details of the interventions offered for LBP managed in the included apps are summarized in Table 3.

**Table 3:** Number and percentage of specific component for low back pain interventions used in included apps.

Specific components	Value, n (%)
Understanding LBP^a^ mechanisms	17 (24.6)
Staying active	9 (13.0)
Postural therapy	8 (11.6)
Lifestyle advice	2 (2.9)
Electrotherapy	3 (4.3)
Cold and heat therapy	8 (11.6)
Manual therapy	4 (5.8)
Medication use	9 (13.0)
Core stability exercise	32 (46.4)
Muscle strengthening	42 (60.9)
Muscle stretching	51 (73.9)
McKenzie exercise	3 (4.3)
Aerobic exercise	8 (11.6)
Yoga	19 (27.5)
Mindfulness	3 (4.3)
Meditation	4 (5.8)
CBT^b^	4 (5.8)
Acupuncture	3 (4.3)

**^a^**LBP: low back pain.

^b^CBT: cognitive behavioral therapy.

### Theoretical and Risk-Related Approaches

None of the 69 apps included in this review referred to or explained their theoretical care model. Only 1 app considered or incorporated a tailored approach to their intervention. A total of 18 (26%) of these apps provided an intervention program based on principles of gradual intervention progression. No app mentioned the age group for which their intervention content was appropriate and none set an age limit for their use. Even fewer, 11 (16%) apps offered safety checks for app users, including identifying red flags, signs, and symptoms of LBP that required medical attention or providing safety-netting advice if the back pain did not resolve or worsen (Multimedia Appendix 3).

## Discussion

### Intervention Content in LBP Apps

We attempted to benchmark the content of the included apps against the most recently published best practice guideline from NICE and found that most LBP self-management app components were consistent with those recommended by NICE. The NICE LBP guidelines [29] recommended the use of a group exercise program, including biomechanical, aerobic, mind-body, or a combination of approaches. Accordingly, muscle stretching, strengthening, core stability exercises, and yoga are the most common interventions in self-management apps. The findings of this review support a 2016 systematic review, which found that interventions in LBP selected by app developers were primarily based on clinical practice guidelines [31]. Similarly, the 2021 Cochrane review also reported moderate-certainty evidence suggesting that different types of exercise therapy are effective in treating LBP [32]. This means that most current LBP self-management apps offer evidence-based interventions.

However, while the content of most apps appears evidence-based, they are not often delivered in the context of a complex intervention and as such do not reflect the current LBP care models. We found that most self-management apps rely on exercise interventions, and very few apps incorporate social and psychological interventions for managing LBP. This suggests that current self-management apps emphasize a more biological care model to manage LBP rather than considering the influence of psychological and social factors in the development and maintenance of pain [33]. Research has emphasized interrelationships among biological changes, psychological status, and the sociocultural context and as such, they all need to be considered to understand the impact of chronic pain and its subsequent management [34]. The biopsychosocial model has been widely accepted as a holistic approach to increase efficacy and outcomes in managing chronic LBP [2,35]. Also, a complex multidisciplinary approach with a biopsychosocial model has been recommended in the early stages of LBP to reduce the likelihood of chronicity following acute LBP [36]. Thus, digital self-management interventions for LBP should consider adopting this model.

### Quality Assessment in Self-Management Apps

Generally, apps for the self-management of LBP are of poor quality as assessed by MARS. Functionality (mean 3.0, SD 0.55) was the domain that scored the highest on the MARS test, as described by other authors [31,37]. This implies that the apps are functioning well, easy to learn and navigate, and efficient. However, the quality assessment revealed that these apps had low scores on the domains of “engagement” (mean 2.1, SD 0.58) and “aesthetics” (mean 2.6, SD 0.61). This indicates that the features that make the app equally engaging and important to a wide user base may have been overlooked. This was partly because most apps did not consider using specific strategies to increase involvement and aesthetics from the user’s point of view (eg, entertainment, interactivity, customization, layout, and graphics). Involving patient users during the development of these apps might better identify their needs and characteristics, and increase adherence to improve self-management and health outcomes [38].

The lowest score on information (mean 2.1, SD 0.46) indicated weakness in the quality and trustworthiness of information presented in the included apps. This was evidenced through assessing credibility (MARS item 18) and evidence base (MARS item 19). In terms of MARS item 18, most apps were developed by either commercial businesses with a vested interest or a legitimate source without verification (eg, has no web page), yet few are developed by credible health organizations (eg, government or universities). The lack of health professional involvement is a consistent issue highlighted by Rizwana, who has expressed concern about the accuracy and trustworthiness of in-app information [39]. In addition, none of the apps available for LBP management were evaluated using a randomized controlled trial. This was evident in MARS item 19, which assesses whether the app has been trialed or tested in the published scientific literature, and therefore the effectiveness and safety of these apps remain unknown. These results align with previous reviews on mHealth apps directed at pain management, in which a lack of scientific basis of the outcomes was found to support the use of such apps [40,41]. A possible explanation could be that most of the apps are of commercial rather than scientific origin, which suggests that the need to promote cross-disciplinary collaboration between academic and commercial institutions might help develop the evidence base for using such apps [37,40].

### Theoretical and Risk-Related Approaches

Current self-management apps do not appear to have used a theoretical rationale in their development. This aligns with the findings from a 2018 review, which found the development of current self-management mHealth apps for patient education programs lacks the support of underpinning theory or framework [42]. An underpinning theoretical model is widely recognized as a crucial component of health interventions and is important when trying to understand key components of the intervention, how they interact, and the mechanisms of the intervention [43-45]. Systematic reviews of existing evidence demonstrate that interventions underpinned by theory are more effective than those that are not [46,47]. Additionally, as a complex multifactorial condition, the management of LBP should consider theory development and identifying the evidence base in accordance with the MRC framework for complex interventions [48].

Regarding personalized care, almost all included apps provided limited customized service. Tailored communications provide individuals with information that is relevant to them and that fits their situation. This can lead to increased perceived personal relevance, user engagement, more in-depth processing of information, and consequently, more desire and motivation to engage in the health behavior change [49]. The importance of personalization of mHealth apps was emphasized in a qualitative review in 2019, which expressed that mHealth apps should meet patients’ needs since they were created for use [22]. Also, our review suggests that the principle of gradual progression from simple to more advanced levels of intervention is not universal. Providing tools that help the user implement exercise progression ensures that the intervention progressively becomes more challenging to continually stimulate adaptations and maintain interest in the program [24]. The Coventry, Aberdeen, and London—Refined (CALO-RE) taxonomy of behavior change techniques highlighted the importance of setting graded tasks, breaking targeted behaviors into smaller, more manageable tasks, and facilitating progress in small increments [50].

Current apps do not evaluate the age limitation of intervention content for which LBP advice and treatments are appropriate, which may pose some risks for the users. Although age ratings in the App Store are often reported, it is based primarily on the degree to which an app contains sensitive information rather than on the applicability of an intervention to different age groups. It is also worth noting that the current NICE guideline for LBP published in 2016 is based on an adult population. A review from the Lancet showed that the evidence underpinning LBP guidelines is drawn almost exclusively from clinical trials of adults, and there are few trials on the treatment of back pain in children [51]. Thus, adult LBP care pathways may not be suitable for adolescents and children [52,53]. When applying these LBP management apps to nonadults, their scope of application needs to be considered. In addition, most included apps performed poorly in providing safety considerations. Safety check advice, including information on the natural history of the illness, advice on worrying symptoms to watch out for, and specific information on how and when to seek help, as well as advice about the follow-up of investigations and hospital referrals, can effectively address uncertainty in the process of LBP management [54]. A scoping review in 2020 discussed that safety concerns within apps were a primary concern [18]. Thus, such approaches will improve the likelihood of providing users with appropriate care and reduce clinical risks associated with self-management [55].

### Evaluation Tool for LBP Apps

Although some self-management apps show relatively high scores on the MARS score, such as “Back Doctor/Pain Relief-1.03.24,” “Perfect Posture&Healthy back-1.5.2,” and “Back pain exercise at home-1.0.99,” they perform poorly on the theoretical and risk-related framework, such as the personalization of care and the age group targeted. Conversely, some self-management apps (eg, BackTrainer-2.0) adopted a tailored approach and addressed risk-related issues, but MARS’ quality assessment scores were not high. Additionally, MARS has not effectively evaluated or included the biopsychosocial care model commonly used in chronic pain [34]. This indicates that current evaluation tools may be limited in their ability to assess LBP self-management apps comprehensively, and consequently, further study is needed to explore whether a holistic tool to evaluate LBP self-management apps is required.

### Barriers to Holistic Digital Apps

Despite the overwhelming evidence recommending the use of holistic multidisciplinary interventions based on a biopsychosocial model for the self-management of LBP, the proliferation of back pain apps on the market that use simple interventions is concerning. It is hard to speculate why this is the case given the strong evidence for the use of a biopsychosocial model in the management of LBP. This may relate to current regulatory approvals. For an app to deliver a complex biopsychosocial intervention it would be classified as a medical device requiring such approvals. Further work is needed to explain why there are so many apps on the market that fail to address the model of care we seek in medical practice, and how current regulatory processes affect this.

Moreover, digital health interventions have been strongly advocated for and implemented in other domains and countries. Notably, draft guidance from NICE has informed digital programs using multidisciplinary models to assist the NHS in delivering specialized services for weight management [56]. Furthermore, the Federal Institute for Drugs and Medical Devices in Germany has authorized the entry of multidisciplinary health care apps with robust trial data into the market, with the overarching objective of empowering clinicians to prescribe health care apps for their patients [57,58]. This illustrates the feasibility of developing multidisciplinary mHealth apps for health care practitioners to recommend. However, it remains imperative to conduct a comprehensive evaluation of the apps using clinical studies to ascertain their efficacy and suitability for widespread prescription.

### Limitations

Apps not specifically targeted for the self-management of LBP (eg, chronic pain, pain management, or MSK apps) were excluded from this review, which may result in potentially eligible apps being missed. While many LBP apps in different countries are available in app stores, our search was limited to the UK iTunes and Google Play stores for practical reasons relating to data-capturing capacity. Therefore, this review did not include apps exclusively available in other countries, and therefore may not represent the broader landscape relating to digital approaches for management of LBP. Only 1 independent reviewer assessed app quality using MARS and discussed uncertainties with 2 other research members until a consensus was reached, which may impact the reliability of the assessments.

### Conclusions

In this review, we identified 69 apps related to the self-management of LBP and rated them using MARS. Most apps scored poor quality due to their approaches to engagement and information, and many emerged as tools for delivering passive information rather than active management apps. Most apps were aligned with guideline-based care. However, no app offered a holistic self-management intervention approach incorporating a biopsychological model. Most apps underestimated the importance of theoretical and risk-related aspects. Thus, current self-management apps for LBP are limited in what they offer.

## Appendix

Multimedia Appendix 1 Characteristics of apps for the self-management of low back pain.

Multimedia Appendix 2 The Mobile App Rating Scale overall and subscale scores.

Multimedia Appendix 3 The content and approach for low back pain interventions used in the included apps.

## Abbreviations

CALO-RE: Coventry, Aberdeen, and London—Refined
LBP: low back pain
MARS: Mobile App Rating Scale
mHealth: mobile health
MRC: Medical Research Council
MSK: musculoskeletal
NICE: National Institute for Health and Care Excellence

## Data Availability

The data sets generated and analyzed during this study are available from the corresponding author on reasonable request.
