# Supplementary material for: mHealth Apps for the Self-Management of Low Back Pain: Systematic Search in App Stores and Content Analysis
Source: JMIR Mhealth Uhealth. 2024 Feb 1;12:e53262. doi: 10.2196/53262 (PMC10870204; doi:10.2196/53262)
Supplement: Multimedia Appendix 1 [file mhealth_v12i1e53262_app1.doc]

| **N** | **App Name–version** | **Developer** | **Age Rating** | **Star ratings** | **Update Date** | **Platform** | **Price (GBP)** | **Delivery** |
| --- | --- | --- | --- | --- | --- | --- | --- | --- |
| 1 | Back pain exercise at home-1.0.99 | Vladimir Ratsev | 4+ | 4.8 | 08/21 | iOS | Free | Video and text description |
| 2 | MSK Help-2.1.0 | NHS 24 | 12+ | 3.7 | 11/20 | iOS | Free | Video |
| 3 | Back Doctor/Pain Relief-1.03.24 | Robert Watkins | 12+ | 4.7 | 10/17 | iOS | Free | Video and text description |
| 4 | Back Pain Yoga SSA-1.0.1 | Yaroslav Petrov | 4+ | 4.1 | 10/18 | iOS | Free | Video |
| 5 | Atlas Low Back Pain-1.0.2 | Atlas Health Group | 4+ | — | 10/21 | iOS | Free | Picture and text description |
| 6 | 6 Minute Back Pain Relief-1.3 | Sunset Games | 4+ | 3.0 | 05/19 | iOS | Free | Video |
| 7 | Lower back Pain Exercises-2.1 | Stefan Roobol | 4+ | 2.0 | 11/19 | iOS | 1.79 | Video |
| 8 | The Truth About Low Back Pain-1.1 | Clinically Relevant Technologies | 12+ | 5.0 | 03/18 | iOS | Free | Video and text description |
| 9 | AudioFysio Lower Back Pain App-1.0.19 | PM Health | 4+ | — | 10/21 | iOS | 2.99* | Video |
| 10 | Yoga for Back Pain Relief-2.2.5 | Saagara | 12+ | 2.6 | 08/17 | iOS | 2.49 | Video |
| 11 | The Back to Health App-1.21 | Healthcare Technologies | 17+ | 5.0 | 01/22 | iOS | 9.99* | Video and text description |
| 12 | Back Workout & Correct Posture-4.0 | Vladyslav Verbytskyi | 4+ | — | 05/21 | iOS | Free | Video |
| 13 | Lower Back Yoga-2.5.0 | Centre de Yoga - La Source SARL | 4+ | 4.5 | 01/18 | iOS | Free | Video |
| 14 | Perfect Posture & Healthy back-1.5.2 | Sergo Kupreishvili | 4+ | 4.9 | 01/22 | iOS | 2.99* | Picture and text description |
| 15 | 10 Min Lower Back Therapy Workout Challenge-1.5 | Mobway Soluations SRL | 4+ | 4.2 | 05/18 | iOS | Free | Picture and text description |
| 16 | Heal Your Back-1.0.2 | Wolfgang Saiger | 4+ | 4.7 | 10/20 | iOS | 3.49* | Video |
| 17 | Posture-training for back-2.1.2 | Alexander Senin | 4+ | 4.7 | 08/18 | iOS | 4.49* | Video and text description |
| 18 | Lower Back Challenge Workout-2.1 | Mobway Solution SRL | 4+ | — | 01/22 | iOS | Free | Picture |
| 19 | Protect Your Back - Tour Tempo-1.9 | Tour Tempo | 4+ | 4.7 | 05/21 | iOS | 17.99 | Video |
| 20 | BackTrainer-2.0 | Ihanwel.com | 4+ | 5.0 | 11/19 | iOS | 8.99 | Video, picture, and text description |
| 21 | 5 Minutes Back Workout at Home-1.1 | Yash Sachapara | 4+ | — | 02/21 | iOS | Free | Text description |
| 22 | BackBetter-1.0 | Michael Simon Baliey | 4+ | 5.0 | — | iOS | Free | Picture |
| 23 | Low Back Care-1.0 | Equipped Motion | 17+ | 5.0 | — | iOS | 0.89 | Video and text description |
| 24 | Healthy Spine Straight Posture-1.2.0 | Nexoft Yazilim Limited Sirketi | 4+ | 5.0 | 02/22 | iOS | Free | Video and text description |
| 25 | Back Pain Relief-1.0 | Samantha Roobol | 4+ | — | — | iOS | Free | Picture and text description |
| 26 | Lower Back Pain and Sciatica Relief Exercises-4.2.4 | App4Life dev | PEGI 3 | 4.3 | 05/20 | Android | Free | Video and text description |
| 27 | Lower Back Pain Exercises-1.5 | 1B Studio Ltd | PEGI 3 | 3.8 | 07/22 | Android | Free | Picture |
| 28 | Back Pain Relief Yoga at Home-8.0 | Dr. Zio-Yoga Teacher | PEGI 3 | 4.4 | 03/23 | Android | Free | Video and text description |
| 29 | Back Pain Relief Exercises-1.2 | 1B Studio Ltd | PEGI 3 | — | 03/21 | Android | Free | Picture and text description |
| 30 | Back Pain Relief-1.0 | Pro Learning Apps | PEGI 3 | 5.0 | 12/19 | Android | Free | Picture and text description |
| 31 | Back Pain Relief Exercise Home-1.0.2 | Gym Fitness Technology | PEGI 3 | 4.3 | 01/21 | Android | Free | Video |
| 32 | 6 Minute Back Pain Relief-3.7 | Round1Fight | PEGI 3 | 4.5 | 05/19 | Android | Free | Video |
| 33 | Exercises for lower back pain-3.20.3.7 | adminapps | PEGI 3 | 3.4 | 10/21 | Android | Free | Picture and text description |
| 34 | Healthy Spine&Straight Posture-3.0.2 | Nexoft - Fitness Apps | PEGI 3 | 4.8 | 05/23 | Android | 3.59* | Video and text description |
| 35 | Back Pain-1.1 | Health care IT | PEGI 3 | — | 03/20 | Android | Free | Text description |
| 36 | Back Pain Relief Exercises-1.94 | Khobta App | PEGI 3 | 4.2 | 06/23 | Android | Free | Picture and text description |
| 37 | Back Pain - causes, symptoms, treatments-1.0.0 | Cursed Apps | PEGI 3 | 4.2 | 09/17 | Android | Free | Text description |
| 38 | BACK PAIN EXERCISES-1.0 | Supportive Apps | PEGI 3 | — | 03/18 | Android | Free | Picture and text description |
| 39 | Back Pain Relief Yoga Poses-19.1 | Proven Digital Web Solutions | PEGI 3 | 3.5 | 11/20 | Android | Free | Picture and text description |
| 40 | Lower back pain yoga-1.1 | DhadbadatiApps | PEGI 3 | — | 12/20 | Android | Free | Text description |
| 41 | Back Pain Relief Exercises - Get Fit Again-1.4 | Aryn Chadha | PEGI 3 | — | 12/20 | Android | Free | Picture and text description |
| 42 | Back Pain Protocols-0.5 | Dr.Isaac's Holistic Wellness | PEGI 3 | — | 12/18 | Android | Free | Picture and text description |
| 43 | Back Pain Exercises-1.0.1 | Adsum Studios | PEGI 3 | — | 04/18 | Android | Free | Picture and text description |
| 44 | Back Pain Exercices-1.0 | vasques.andromo | PEGI 3 | — | 04/21 | Android | Free | Picture and text description |
| 45 | BACK PAIN CAUSES & TREATMENT-1.0 | salim garba usman | PEGI 3 | — | 11/19 | Android | Free | Text description |
| 46 | Lower Back Pain Relief Exercise-1.0 | AppsFriendly | PEGI 3 | 4.3 | 12/19 | Android | Free | Education and Text description |
| 47 | Stretching Exercises for Back Pain-3.7.1.2.1 | tbeapps | PEGI 3 | 3.7 | 03/19 | Android | Free | Picture and text description |
| 48 | Yoga Poses for Back Pain-1.0 | Expert Lab Team | PEGI 3 | 4.1 | 10/17 | Android | Free | Picture and text description |
| 49 | Back Pain Guide-1.3 | Everyone Learning Apps | PEGI 3 | — | 05/20 | Android | Free | Text description |
| 50 | Abs, Core & Back Workout at Home-1.0 | Fitify Apps | PEGI 3 | 4.7 | 08/21 | Android | Free | Video |
| 51 | Back Workout & Exercises by Fitness Coach-1.0.14 | FitStar Apps s.r.o. | PEGI 3 | 4.7 | 12/20 | Android | Free | Video |
| 52 | Treat Back Pain-1.0.8 | Nature Healthy Care | PEGI 3 | — | 06/23 | Android | Free | Text description |
| 53 | Back Pain Exercise For All-1.0.2 | Creative Apps Here | PEGI 3 | — | 01/21 | Android | Free | Picture and text description |
| 54 | Back Pain & How To Prevent It-44.0 | Twayesh Projects | PEGI 3 | 3.7 | 08/18 | Android | Free | Text description |
| 55 | Sciatic Nerve Pain Exercises-3.20.0.5 | adminapps | PEGI 3 | 3.5 | 08/21 | Android | Free | Picture and text description |
| 56 | Sciatica Pain Exercises-3.8.1.3.1 | abayapps | PEGI 3 | 3.6 | 07/19 | Android | Free | Picture and text description |
| 57 | Sciatica Pain Exercises-3.20.3.4 | tbeapps | PEGI 3 | 3.5 | 10/21 | Android | Free | Picture and text description |
| 58 | Back Pain Tips-1.3 | Galaxy Studio Digital | PEGI 3 | — | 05/20 | Android | Free | Text description |
| 59 | Back Posture Correction Yoga-8.8 | Dr. Zio - Yoga Teacher | PEGI 3 | 4.0 | 03/23 | Android | Free | Video and text description |
| 60 | Back Workout & Back Pain App by Fitstar-9.0 | ANIJ | PEGI 3 | — | 04/20 | Android | Free | Picture and text description |
| 61 | Back Pain Relief-1.0.6 | Powerpunch - data driven workout programs | PEGI 3 | — | 04/23 | Android | 4.29* | Video, picture and text description |
| 62 | Low back pain exercises-3.20.3.3 | tbeapps | PEGI 3 | — | 10/21 | Android | Free | Picture and text description |
| 63 | Back Workout & Correct Posture-6.5.5 | VerbLike Fitness Group | PEGI 3 | — | 03/23 | Android | Free | Picture and text description |
| 64 | Lower Back Pain Relief-1.1 | How to tips and tricks | PEGI 3 | — | 06/22 | Android | Free | Text description |
| 65 | Relief of back pain exercises-1.0 | D'isla Games | PEGI 3 | — | 05/23 | Android | Free | Text description |
| 66 | Back pain Precautions-5.1 | Smartrehab | PEGI 3 | — | 06/23 | Android | Free | Picture and text description |
| 67 | Low Back Pain Rehabilitation E-1.4 | vorgueapps | PEGI 3 | — | 01/20 | Android | Free | Picture and text description |
| 68 | Straight Posture-Healthy Spine-3.4.8 | mEL Studio | PEGI 3 | 4.7 | 05/23 | Android | 4.99* | Video, picture and text description |
| 69 | Back Pain Relief Exercises-1.1.2 | World Gym Fitness JS | PEGI 3 | — | 11/22 | Android | Free | Video and picture |

* Presence of in-app purchases
