# Supplementary material for: mHealth Apps for the Self-Management of Low Back Pain: Systematic Search in App Stores and Content Analysis
Source: JMIR Mhealth Uhealth. 2024 Feb 1;12:e53262. doi: 10.2196/53262 (PMC10870204; doi:10.2196/53262)
Supplement: Multimedia Appendix 2 [file mhealth_v12i1e53262_app2.doc]

| N | App Name | MARS Engagement | MARS Functionality | MARS  Aesthetics | MARS  Information | MARS  Total score |
| --- | --- | --- | --- | --- | --- | --- |
| 1 | Back pain exercise at home | 3.0 | 4.0 | 3.3 | 2.9 | 3.30 |
| 2 | MSK Help | 3.0 | 4.3 | 4.3 | 2.9 | 3.61 |
| 3 | Back Doctor / Pain Relief | 2.4 | 4.0 | 3.7 | 3.1 | 3.30 |
| 4 | Back Pain Yoga SSA | 2.2 | 3.8 | 2.7 | 2.1 | 2.69 |
| 5 | Atlas Low Back Pain | 2.4 | 3.5 | 3.0 | 2.1 | 2.76 |
| 6 | 6 Minute Back Pain Relief | 2.0 | 2.8 | 2.7 | 2.4 | 2.46 |
| 7 | Lower back Pain Exercises | 3.0 | 3.5 | 3.7 | 2.1 | 3.08 |
| 8 | The Truth About Low Back Pain | 3.0 | 3.8 | 3.0 | 2.7 | 3.12 |
| 9 | AudioFysio Lower Back Pain App | 1.8 | 3.3 | 3.0 | 2.6 | 2.66 |
| 10 | Yoga for Back Pain Relief | 2.8 | 2.8 | 2.3 | 2.3 | 2.54 |
| 11 | The Back to Health App | 2.4 | 2.5 | 2.7 | 2.1 | 2.43 |
| 12 | Back Workout & Correct Posture | 3.6 | 3.5 | 3.0 | 2.0 | 3.03 |
| 13 | Lower Back Yoga | 1.8 | 3.0 | 2.7 | 2.3 | 2.44 |
| 14 | Perfect Posture & Healthy back | 3.4 | 3.5 | 3.7 | 2.7 | 3.32 |
| 15 | 10 Min Lower Back Therapy Workout Challenge | 2.6 | 2.3 | 2.0 | 1.4 | 2.07 |
| 16 | Heal Your Back | 2.0 | 2.5 | 3.3 | 2.4 | 2.57 |
| 17 | Posture-training for back | 2.2 | 3.0 | 2.7 | 2.0 | 2.47 |
| 18 | Lower Back Challenge Workout | 2.4 | 2.3 | 2.0 | 1.3 | 1.98 |
| 19 | Protect Your Back - Tour Tempo | 1.6 | 3.0 | 2.0 | 2.9 | 2.36 |
| 20 | BackTrainer | 2.2 | 3.3 | 3.3 | 2.4 | 2.80 |
| 21 | 5 Minutes Back Workout at Home | 2.2 | 3.0 | 3.0 | 2.0 | 2.55 |
| 22 | BackBetter | 2.2 | 3.8 | 3.7 | 2.4 | 3.01 |
| 23 | Low Back Care | 1.8 | 2.0 | 2.0 | 1.6 | 1.84 |
| 24 | Healthy Spine Straight Posture | 2.6 | 2.8 | 2.0 | 2.6 | 2.48 |
| 25 | Back Pain Relief | 2.2 | 3.0 | 3.7 | 2.9 | 2.93 |
| 26 | Lower Back Pain and Sciatica Relief Exercises | 2.2 | 3.3 | 3.0 | 1.9 | 2.58 |
| 27 | Lower Back Pain Exercises | 2.4 | 3.5 | 2.3 | 1.6 | 2.45 |
| 28 | Back Pain Relief Yoga at Home | 2.6 | 2.5 | 2.7 | 2.4 | 2.55 |
| 29 | Back Pain Relief Exercises | 2.4 | 2.5 | 2.3 | 2.1 | 2.34 |
| 30 | Back Pain Relief | 1.6 | 3.0 | 2.3 | 2.0 | 2.23 |
| 31 | Back Pain Relief Exercise Home | 1.6 | 2.8 | 2.0 | 2.1 | 2.12 |
| 32 | 6 Minute Back Pain Relief | 2.2 | 2.3 | 2.7 | 2.1 | 2.31 |
| 33 | Exercises for lower back pain | 1.6 | 3.0 | 2.0 | 2.1 | 2.19 |
| 34 | Healthy Spine & Straight Posture - Back exercises | 3.6 | 3.0 | 3.3 | 2.7 | 3.16 |
| 35 | Back Pain | 1.6 | 2.8 | 2.0 | 2.1 | 2.12 |
| 36 | Back Pain Relief Exercises | 2.2 | 3.5 | 2.3 | 1.7 | 2.44 |
| 37 | Back Pain - causes, symptoms, treatments | 1.6 | 3.5 | 3.0 | 1.6 | 2.42 |
| 38 | BACK PAIN EXERCISES | 1.6 | 3.3 | 2.0 | 1.9 | 2.18 |
| 39 | Back Pain Relief Yoga Poses | 1.6 | 3.8 | 2.0 | 2.4 | 2.44 |
| 40 | Lower back pain yoga | 1.2 | 2.3 | 2.3 | 1.6 | 1.84 |
| 41 | Back Pain Relief Exercises - Get Fit Again | 1.8 | 3.5 | 2.3 | 2.4 | 2.52 |
| 42 | Back Pain Protocols | 1.6 | 2.3 | 1.7 | 1.9 | 1.84 |
| 43 | Back Pain Exercises | 1.4 | 2.0 | 1.0 | 1.1 | 1.39 |
| 44 | Back Pain Exercices | 1.8 | 3.5 | 2.0 | 1.7 | 2.25 |
| 45 | BACK PAIN CAUSES & TREATMENT | 1.6 | 3.8 | 2.7 | 1.6 | 2.40 |
| 46 | Lower Back Pain Relief Exercise | 3.2 | 3.5 | 3.0 | 2.0 | 2.93 |
| 47 | Stretching Exercises for Back Pain | 2.2 | 3.5 | 3.0 | 2.0 | 2.68 |
| 48 | Yoga Poses for Back Pain | 2.2 | 3.5 | 2.0 | 1.6 | 2.32 |
| 49 | Back Pain Guide | 1.6 | 3.5 | 2.7 | 1.9 | 2.41 |
| 50 | Abs, Core & Back Workout at Home-1.0 | 2.6 | 2.8 | 3.0 | 2.1 | 2.62 |
| 51 | Back Workout & Exercises by Fitness Coach | 2.6 | 3.0 | 2.7 | 2.4 | 2.67 |
| 52 | Treat Back Pain | 1.4 | 2.3 | 2.0 | 1.1 | 1.70 |
| 53 | Back Pain Exercise For All | 1.8 | 3.0 | 2.3 | 2.1 | 2.32 |
| 54 | Back Pain & How To Prevent It | 1.6 | 3.0 | 2.0 | 1.6 | 2.04 |
| 55 | Sciatic Nerve Pain Exercises | 2.0 | 3.0 | 2.7 | 2.6 | 2.56 |
| 56 | Sciatica Pain Exercises | 1.6 | 2.8 | 3.0 | 2.0 | 2.34 |
| 57 | Sciatica Pain Exercises | 1.6 | 3.0 | 2.0 | 2.4 | 2.26 |
| 58 | Back Pain Tips | 1.6 | 2.3 | 2.0 | 1.6 | 1.86 |
| 59 | Back Posture Correction Yoga | 3.2 | 3.3 | 3.0 | 2.9 | 3.08 |
| 60 | Back Workout & Back Pain App by Fitstar | 1.6 | 3.0 | 2.7 | 1.6 | 2.21 |
| 61 | Back Pain Relief | 1.6 | 2.3 | 2.0 | 2.1 | 2.00 |
| 62 | Low back pain exercises | 2.0 | 2.8 | 2.3 | 1.7 | 2.20 |
| 63 | Back Workout & Correct Posture | 2.2 | 3.0 | 2.7 | 1.7 | 2.40 |
| 64 | Lower Back Pain Relief | 1.2 | 2.0 | 2.0 | 1.4 | 1.66 |
| 65 | Relief of back pain exercises | 1.6 | 2.0 | 1.7 | 1.6 | 1.71 |
| 66 | Back pain Precautions | 1.8 | 2.8 | 2.7 | 2.1 | 2.34 |
| 67 | Low Back Pain Rehabilitation E | 1.6 | 2.3 | 1.7 | 2.3 | 1.95 |
| 68 | Straight Posture-Healthy Spine | 3.2 | 3.3 | 3.0 | 2.6 | 3.01 |
| 69 | Back Pain Relief Exercises | 2.0 | 2.5 | 2.0 | 1.7 | 2.05 |
| Mean score | | 2.1 | 3.0 | 2.6 | 2.1 | 2.4 |
| SD | | 0.58 | 0.55 | 0.61 | 0.46 | 0.44 |
