# Supplementary material for: mHealth Apps for the Self-Management of Low Back Pain: Systematic Search in App Stores and Content Analysis
Source: JMIR Mhealth Uhealth. 2024 Feb 1;12:e53262. doi: 10.2196/53262 (PMC10870204; doi:10.2196/53262)
Supplement: Multimedia Appendix 3 [file mhealth_v12i1e53262_app3.doc]

| **N** | **app Name–version** | **Type of component** | **Specific content** | **Theoretical framework** | | | **Risk-related framework** | |
| --- | --- | --- | --- | --- | --- | --- | --- | --- |
|  |  |  |  | **Theoretical care model** | **Personalisation of care** | **Intervention of progression approach** | **Age group targeted** | **Safety check** |
| 1 | Back pain exercise at home-1.0.99 | Exercise programme | Strengthening exercise, Stretching exercise, Core stability exercise |  |  |  |  |  |
| 2 | MSK Help-2.1.0 | Exercise programme+Patient education | CLBP mechanisms, Good posture, Staying active, Cold and heat therapy, Medication use |  |  | √ |  |  |
| 3 | Back Doctor/Pain Relief-1.03.24 | Exercise programme+Patient education+Psychological intervention | Strengthening exercise, Stretching exercise, Core stability exercise, Aerobic exercises, Staying active, Good posture, Lifestyle, Diet, Mindfulness, Relaxation, Massage |  |  | √ |  |  |
| 4 | Back Pain Yoga SSA-1.0.1 | Exercise programme | Yoga |  |  | √ |  |  |
| 5 | Atlas Low Back Pain-1.0.2 | Exercise programme+Patient education | Strengthening exercise, Stretching exercise, Core stability exercise, Avoiding bed rest, Staying active |  |  | √ |  |  |
| 6 | 6 Minute Back Pain Relief-1.3 | Exercise programme | Strengthening exercise, Stretching exercise, Yoga |  |  |  |  |  |
| 7 | Lower back Pain Exercises-2.1 | Exercise programme | Strengthening exercise, Stretching exercise, Yoga |  |  |  |  |  |
| 8 | The Truth About Low Back Pain-1.1 | Patient education | CLBP mechanisms, Keeping moving, Staying active, Thinking positive, Carefully medication use |  |  |  |  | √ |
| 9 | Bella’s Lower Back Pain app-1.0.19 | Exercise programme | Strengthening exercise, Stretching exercise, Core stability exercise |  |  |  |  |  |
| 10 | Yoga for Back Pain Relief-2.2.5 | Exercise programme | Yoga |  |  | √ |  |  |
| 11 | The Back to Health app-1.21 | Exercise programme+Psychological intervention | Strengthening exercise, Stretching exercise, Core stability exercise, Meditation |  |  | √ |  |  |
| 12 | Back Workout&Correct Posture-4.0 | Exercise programme | Strengthening exercise, Stretching exercise |  |  | √ |  |  |
| 13 | Lower Back Yoga-2.5.0 | Exercise programme | Yoga |  |  |  |  |  |
| 14 | Perfect Posture&Healthy back-1.5.2 | Exercise programme | Strengthening exercise, Stretching exercise |  |  | √ |  |  |
| 15 | 10 Min Lower Back Therapy Workout Challenge-1.5 | Exercise programme | Strengthening exercise, Stretching exercise |  |  |  |  |  |
| 16 | Heal Your Back-1.0.2 | Exercise programme | Strengthening exercise, Stretching exercise |  |  |  |  |  |
| 17 | Posture-training for back-2.1.2 | Exercise programme | Strengthening exercise |  |  | √ |  |  |
| 18 | Lower Back Challenge Workout-2.1 | Exercise programme | Strengthening exercise, Stretching exercise, Core stability exercise |  |  |  |  |  |
| 19 | Protect Your Back - Tour Tempo-1.9 | Exercise programme+Patient education | Spine and core work mechanism, Strengthening exercise, Stretching exercise, Core stability exercise |  |  | √ |  |  |
| 20 | BackTrainer-2.0 | Exercise programme+Patient education | Spine anatomy, Significance of trunk muscle, Strengthening exercise, Stretching exercise, Core stability exercise |  |  | √ |  | √ |
| 21 | 5 Minutes Back Workout at Home-1.1 | Exercise programme | Strengthening exercise, Core stability exercise |  |  | √ |  |  |
| 22 | BackBetter-1.0 | Exercise programme+ Psychological intervention | Strengthening exercise, Stretching exercise, Core stability exercise, Meditation |  |  | √ |  |  |
| 23 | Low Back Care-1.0 | Exercise programme+Patient education | Core stability exercise, CLBP introduction |  |  |  |  |  |
| 24 | Healthy Spine Straight Posture-1.2.0 | Exercise programme | Strengthening exercise, Stretching exercise, Core stability exercise, Yoga |  |  | √ |  |  |
| 25 | Back Pain Relief-1.0 | Exercise programme | Strengthening exercise, Stretching exercise, Core stability exercise, Yoga |  |  | √ |  |  |
| 26 | Lower Back Pain and Sciatica Relief Exercises | Exercise programme | Strengthening exercise, Stretching exercise, Core stability exercise, McKenzie exercise |  |  |  |  |  |
| 27 | Lower Back Pain Exercises | Exercise programme | Stretching exercise |  |  |  |  |  |
| 28 | Back Pain Relief Yoga at Home | Exercise programme | Strengthening exercise, Stretching exercise, Core stability exercise, McKenzie exercise, Yoga |  |  |  |  |  |
| 29 | Back Pain Relief Exercises | Exercise programme | Stretching exercise |  |  |  |  |  |
| 30 | Back Pain Relief | Exercise programme+Patient education | Stretching exercise, Core stability exercise, CLBP mechanisms |  |  |  |  | √ |
| 31 | Back Pain Relief Exercise Home | Exercise programme | Stretching exercise |  |  |  |  |  |
| 32 | 6 Minute Back Pain Relief | Exercise programme | Strengthening exercise, Stretching exercise, Yoga |  |  |  |  |  |
| 33 | Exercises for lower back pain | Exercise programme | Strengthening exercise, Stretching exercise |  |  |  |  |  |
| 34 | Healthy Spine & Straight Posture - Back exercises | Exercise programme | Stretching exercise, Core stability exercise |  |  |  |  |  |
| 35 | Back Pain | Patient education | CLBP mechanisms, Staying active |  |  |  |  |  |
| 36 | Back Pain Relief Exercises | Exercise programme | Strengthening exercise, Stretching exercise, Core stability exercise, McKenzie exercise |  |  |  |  |  |
| 37 | Back Pain - causes, symptoms, treatments | Patient education | CLBP mechanisms, Ergonomic education, NSAIDs, Cold and Heat therapy, Ultrasound, TENS, Soft tissue technique, CBT, Yoga, Strengthening exercise, Core stability exercise |  |  |  |  | √ |
| 38 | BACK PAIN EXERCISES | Exercise programme+Patient education | Strengthening exercise, Stretching exercise, Core stability exercise, Aerobic exercises, Mindfulness, Meditation, Staying active, Cold and heat therapy, NSAIDs, Analgesics, Muscle relaxants, Lifestyle modification |  |  |  |  |  |
| 39 | Back Pain Relief Yoga Poses | Exercise programme | Yoga |  |  |  |  |  |
| 40 | Lower back pain yoga | Exercise programme | Yoga |  |  |  |  |  |
| 41 | Back Pain Relief Exercises - Get Fit Again | Exercise programme | Stretching exercise |  |  |  |  |  |
| 42 | Back Pain Protocols | Exercise programme+Patient education | CLBP mechanisms, Strengthening exercise, Stretching exercise, Core stability exercise, Aerobic exercises, Aquatic exercise, Acupuncture, Taichi, Yoga |  |  |  |  | √ |
| 43 | Back Pain Exercises | Exercise programme | Stretching exercise |  |  |  |  |  |
| 44 | Back Pain Exercices | Exercise programme | Strengthening exercise, Stretching exercise, Aerobic exercises |  |  |  |  |  |
| 45 | BACK PAIN CAUSES & TREATMENT | Patient education | CLBP mechanisms, Ergonomic education, NSAIDs, Cold and heat therapy, Electrical stimulation, ultrasound, Traction, CBT, TENS, Acupuncture, Yoga, Avoid bad postures, Stretching exercise, Core stability exercise |  |  |  |  | √ |
| 46 | Lower Back Pain Relief Exercise | Exercise programme | Strengthening exercise |  |  |  |  |  |
| 47 | Stretching Exercises for Back Pain | Exercise programme | Stretching exercise |  |  |  |  |  |
| 48 | Yoga Poses for Back Pain | Exercise programme | Yoga |  |  |  |  |  |
| 49 | Back Pain Guide | Patient education | CLBP mechanisms, Ergonomic education, Avoiding bad postures, Stretching exercise, Core stability exercise |  |  |  |  | √ |
| 50 | Abs, Core & Back Workout at Home-1.0 | Exercise programme | Strengthening exercise, Core stability exercise |  |  |  |  |  |
| 51 | Back Workout & Exercises by Fitness Coach-1.0.14 | Exercise programme | Strengthening exercise, Stretching exercise |  |  | √ |  |  |
| 52 | Treat Back Pain | Patient education | Strengthening exercise, Core stability exercise, Aerobic exercises, Pilates, Cold and heat therapy, Staying active, Returning to Work, NSAIDs, Analgesics, Muscle relaxants, CBT |  |  |  |  |  |
| 53 | Back Pain Exercise For All | Exercise programme | Strengthening exercise, Stretching exercise |  |  |  |  |  |
| 54 | Back Pain & How To Prevent It | Patient education | CLBP mechanisms, Ergonomic education, Analgesics, Muscle relaxants, acupuncture, acupressure, massage, Cold and heat therapy, Stretching exercise, Avoiding bad posture, Staying physically fit |  |  |  |  |  |
| 55 | Sciatic Nerve Pain Exercises | Exercise programme+Patient education | CLBP mechanisms, Stretching exercise |  |  |  |  |  |
| 56 | Sciatica Pain Exercises | Exercise programme | Stretching exercise |  |  |  |  |  |
| 57 | Sciatica Pain Exercises | Exercise programme | Stretching exercise |  |  |  |  |  |
| 58 | Back Pain Tips | Patient education | CLBP mechanisms, NSAIDs, Cold and Heat therapy, Ultrasound, TENS, Soft tissue technique, CBT, Yoga, Strengthening exercise, Core stability exercise |  |  |  |  | √ |
| 59 | Back Posture Correction Yoga | Exercise programme | Yoga |  |  |  |  |  |
| 60 | Back Workout & Back Pain App by Fitstar | Exercise programme | Strengthening exercise |  |  |  |  |  |
| 61 | Back Pain Relief-1.0.6 | Exercise programme | Strengthening exercise, Stretching exercise, Core stability exercise, Aerobic exercises, Yoga |  |  | √ |  |  |
| 62 | Low back pain exercises-3.20.3.3 | Exercise programme | Strengthening exercise, Stretching exercise |  |  |  |  |  |
| 63 | Back Workout & Correct Posture-6.5.5 | Exercise programme | Strengthening exercise, Stretching exercise, Core stability exercise |  |  | √ |  |  |
| 64 | Lower Back Pain Relief-1.1 | Patient education | CLBP mechanisms, Stretching exercise, Painkillers, Cold and heat therapy, Avoiding bad posture, |  |  |  |  |  |
| 65 | Relief of back pain exercises-1.0 | Exercise programme | Strengthening exercise, Stretching exercise, Aerobic exercises |  |  |  |  | √ |
| 66 | Back pain Precautions-5.1 | Patient education | Avoiding bad posture, Strengthening exercise, Stretching exercise, Core stability exercise, Aerobic exercises, Yoga, Mindfulness, Meditation, Ergonomic education |  |  |  |  | √ |
| 67 | Low Back Pain Rehabilitation E-1.4 | Exercise programme | Avoiding bad posture, Strengthening exercise, Stretching exercise, Core stability exercise, Staying active |  |  |  |  | √ |
| 68 | Straight Posture-Healthy Spine-3.4.8 | Exercise programme | Strengthening exercise, Stretching exercise, Core stability exercise |  | √ |  |  |  |
| 69 | Back Pain Relief Exercises-1.1.2 | Exercise programme | Stretching exercise |  |  |  |  |  |
| Total |  |  |  | 0/69 | 1/69 | 18/69 | 0/69 | 11/69 |
